# Impact of age on neurofilament light chain in Friedreich ataxia: a 1-year longitudinal study

**DOI:** 10.1093/braincomms/fcaf331

**Published:** 2025-09-10

**Authors:** Sara Petrillo, Alessia Mongelli, Anna Castaldo, Lidia Sarro, Samuele Azzarelli, Riccardo Ronco, Barbara Castellotti, Cinzia Gellera, Fiorella Piemonte, Caterina Mariotti

**Affiliations:** Unit of Muscular and Neurodegenerative Diseases, Bambino Gesù Children's Hospital, IRCCS, Rome 00165, Italy; Neurology Department, NRSC, Fondazione IRCCS Istituto Neurologico Carlo Besta, Via Celoria 11, Milano 20133, Italy; Neurology Department, NRSC, Fondazione IRCCS Istituto Neurologico Carlo Besta, Via Celoria 11, Milano 20133, Italy; Neurology Department, NRSC, Fondazione IRCCS Istituto Neurologico Carlo Besta, Via Celoria 11, Milano 20133, Italy; Neurology Department, NRSC, Fondazione IRCCS Istituto Neurologico Carlo Besta, Via Celoria 11, Milano 20133, Italy; Neurology Department, NRSC, Fondazione IRCCS Istituto Neurologico Carlo Besta, Via Celoria 11, Milano 20133, Italy; University of Milan, Medical School, via Festa del Perdono, 7, Milano 20122, Italy; Unit of Medical Genetics and Neurogenetics, Fondazione IRCCS Istituto Neurologico Carlo Besta, Milano 20133, Italy; Unit of Medical Genetics and Neurogenetics, Fondazione IRCCS Istituto Neurologico Carlo Besta, Milano 20133, Italy; Unit of Muscular and Neurodegenerative Diseases, Bambino Gesù Children's Hospital, IRCCS, Rome 00165, Italy; Neurology Department, NRSC, Fondazione IRCCS Istituto Neurologico Carlo Besta, Via Celoria 11, Milano 20133, Italy

**Keywords:** Friedreich ataxia, longitudinal study, pediatric population, neurofilament light chain

## Abstract

Friedreich's ataxia (FRDA) is a recessive inherited ataxia caused by intronic GAA repeat expansions in *FXN* gene. The repeat length is the major determinant of age at onset, usually occurring in adolescence. Clinical manifestations include progressive gait and limb ataxia, sensory loss, cardiomyopathy, and scoliosis. Neurofilament light chain protein (NfL) has been recently studied as a potential plasma biomarker for the disease. We performed a longitudinal study in 62 patients with FRDA, including 12 children (age 12–17 years) and 50 adult patients (age 18–45). The characteristics of our patient cohort largely matched those of a population mostly recruited in therapeutical clinical trials, with a mean age of 25.1 ± 8.5 years, age at onset 13.1 ± 4.8 years, and disease duration 12 ± 7 years. We found higher NfL levels in children in comparison with adult patients. Plasma concentrations remained stable at 1-year follow-up. We observed a significantly inverse correlation between plasma NfL levels and patient ages, while no correlations were found with other clinical or genetic variables. Our study confirms the typical NfL profile in FRDA patients. Our data further support the role of NfL as early indicator of axonal damage and as potential pharmacodynamic biomarker of therapeutical response especially valuable in pediatric populations.

## Teaser

Neurofilament-light-chains are elevated in patients with Friedreich Ataxia compared with controls and remain stable after 1 year follow-up. Affected children have higher levels of neurofilament compared with adult patients, reflecting more abundant neurodegeneration in early stages. Our results support plasma neurofilament as a good candidate biomarker for future therapeutic intervention.

## Graphical Abstract

**Graphical Abstract:**
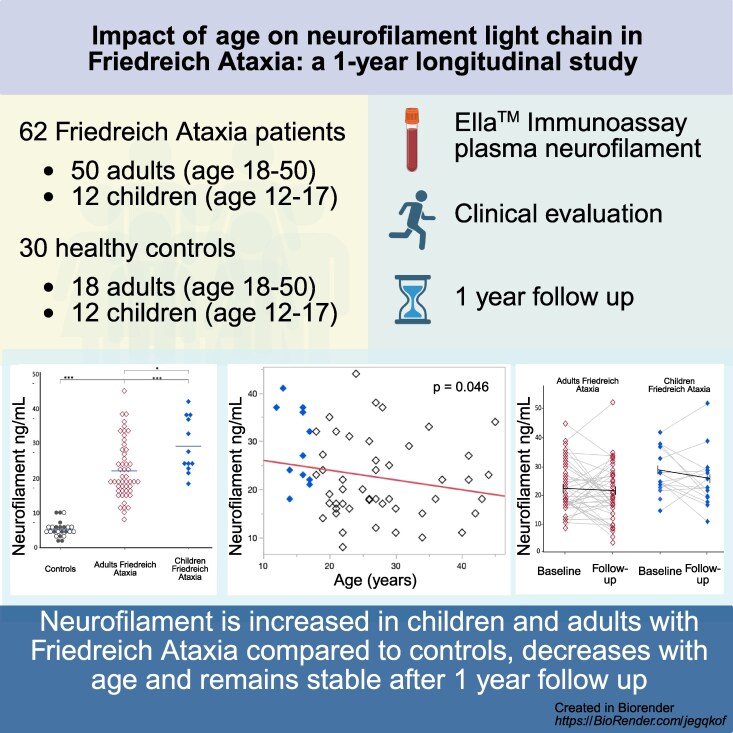

## Introduction

Friedreich's ataxia (FRDA) is an autosomal recessive multi-systemic disorder presenting with progressive gait and limb ataxia, dysarthria, sensory loss, areflexia, cardiomyopathy, diabetes mellitus, and scoliosis.^1^ The onset of symptoms most commonly occurs during childhood or adolescence, and patients become wheelchair-bound on average 15 years after disease onset. The causative genetic defect is a homozygous GAA (guanine-adenine-adenine) trinucleotide repeat expansion in the first intron of the *FXN* gene.^2^ Only few patients (3–4%) are compound heterozygotes for the GAA expansion on one allele and a point mutation on the other allele of *FXN* gene. The presence of GAA expansion leads to reduced amount of both mRNA and encoded protein, frataxin, which is involved in mitochondrial iron metabolism, iron-sulfur cluster biosynthesis, and in the mitochondrial oxidative-phosphorylation system.^3^ Larger expansions correspond to smaller residual amount of frataxin, which in turn determines increased mitochondrial oxidative stress and cellular damage, particularly relevant in the nervous system and the heart. In patients, larger expansions are associated with younger age at onset, more severe phenotype and a more rapid disease progression. In natural history studies and interventional clinical trials FRDA disease progression and response to therapeutical interventions have been commonly tracked by clinical and neuroimaging measures.

In recent years, measurement of neurofilament light chain protein (NfL) has increasingly been considered in the contest of clinical trials and safety monitoring.^4^

NfL is a protein mostly concentrate in neuronal axons, where it plays a crucial role in maintaining stability, shape and regulation of the axon's diameter, while in case of axonal damage, it is released into cerebrospinal fluid and in blood.^5^ NfL levels have been measured in various types of genetic ataxia,^6^ including autosomal dominant spinocerebellar ataxias (SCAs),^7-9^ and FRDA.^10-13^ In patients with SCAs, NfL concentrations are elevated in comparison to healthy controls, and blood levels progressively increase during the course of the diseases. Vice versa, in FRDA patients the NfL concentration is high in the early stage and then it decreases with advancing age. A clear correlation between plasma levels and measures of disease progression has been demonstrated in few studies, though not consistently across all investigations.^10-14^ Here we conducted a 1-year prospective study in a large cohort of FRDA patients, with the aim of comparing NfL level and its profile of change in children and young adults.

## Materials and methods

### Participants and clinical evaluations

We enrolled children (age 12–17 years) and adult patients (age 18 to 45 years) with FRDA. Inclusion criteria were (i) confirmed genetic diagnosis of FRDA, and (ii) age at onset before age 25. Exclusion criteria were the presence of severe concomitant medical/cognitive conditions unrelated to FRDA or clinical conditions preventing the completion of clinical assessments established by the protocol. Healthy control subjects were recruited from the community or from unrelated family members. Study participants were evaluated at baseline and after a 12 ± 2 months interval. Demographical data, clinical and genetic characteristics, and concomitant medical conditions were collected. Neurological examination included administration of SARA scale,^15^ modified FARS scale,^16^ and activity of daily living (ADL) as part of the FARS.^17^

Blood samples were obtained from not fasted participants. The study was evaluated and approved by the Local Ethics Committee on 13 January 2021 (Protocol N. 80). All subjects or legal guardians gave written informed consent for the participation in the study, and all procedures were carried out in accordance with the Declaration of Helsinki.

### NfL and frataxin mRNA analyses

Anonymize blood samples were collected into 5 mL EDTA Vacutainer Tubes (Becton Dickinson, Rutherford, NY) and immediately sent to the laboratory at room temperature. Upon arrival, 3 mL were centrifuged at 2,500×g for 3 min, and the plasma aliquots stored at −80°C until NfL assays. NfL concentrations were measured by the Human Simple Plex assay kit (ProteinSimple, CA, USA) on Ella device (ProteinSimple, CA, USA), according to the manufacturers’ instructions. Calibration of Ella was performed using the in-cartridge factory standard curve, and plasma samples were measured with a 1:2 dilution in Sample Diluent provided by the kit. Triplicates were automatically performed in Simple Plex assay microfluidic platform, and data were expressed as pmol/mL. Plasma obtained from healthy subjects was used as controls to monitor assay performance. Longitudinal samples were tested in the same analytical run.

At baseline, an additional blood sample collection in TempusTM Blood RNA (5 mL) was used for analysis of frataxin mRNA expression. Total RNA was extracted from 3 mL whole blood using Tempus Spin RNA Isolation Reagent kit (ThermoFisher Scientific, Waltham, MA, USA), according to manufacturer’s protocol. One μg RNA samples was reverse transcribed with the LunaScript RT Super mix kit (New England BioLabs, MA, USA). The *FXN* mRNA was measured by qRT-PCR in an ABI PRISM7500 Sequence Detection System (Life Technologies, Carlsbad, CA, United States) using Power SYBR Green I dye chemistry. Data were analyzed using the 2^−ΔΔCt^ method with TATA box binding protein (TBP) as a housekeeping gene and expressed as fold change relative to the controls.

### Statistical analyses

Inter-group differences between two or more groups were assessed using Wilcoxon or Kruskal-Wallis tests. Spearman correlation test was used to correlate NfL levels with clinical and demographical variables. A two-tailed Wilcoxon-Rank test was applied to analyze paired data from longitudinal measurements. Correlation was assessed by computing Spearman ρ. Data are presented as mean ± standard deviation (SD). The level of significance was set at *P* < 0.05. Statistical analyses were conducted using JMP®, version 11 (SAS Institute Inc., USA).

## Results

Between October 2021 and July 2022, we enrolled 62 subjects with FRDA: 12 children (mean age 15.2 ± 1.7 years) and 50 adults (mean age 27.5 ± 7.7). Healthy controls were 12 minors (aged 15.2 ± 1.9 years), and 18 adults (age 26.4 ± 4 years) (Table 1).

**Table 1:** Baseline data of FRDA patients and healthy controls

	FRDA children	Control children	FRDA adults	Control adults
No	12	12	50	18
Sex (F/M)	6/6	6/6	24/26	9/9
Age at exam (years)	15.2 ± 1.7	15.2 ± 1.9	27.5 ± 7.7	26.4 ± 4.0
Age at onset (years)	9.5 ± 2.9	-	14.0 ± 4.9	-
Disease duration (years)	5.9 ± 1.9	-	13.5 ± 7.1	-
SARA score	16.4 ± 4.7	-	20.8 ± 7.6	-
mFARS score	50.4 ± 7.6	-	57.1 ± 14.4	-
ADL score	9.5 ± 5.7	-	15.2 ± 6.3	
NfL (pg/mL)	28.5 ± 7.7**^a^	5.3 ± 2.7	21.6 ± 8.1**	6.3 ± 4.2
FXN mRNA (FXN/TBP)	0.20 ± 0.05**	NA	0.27 ± 0.12**	1.0 ± 0.19

Data are shown as mean ± SD. **P < 0.001 for comparisons between FRDA patients and age–matched controls.

^a^ *P* < 0.05 for comparison between adult and child patients with FRDA (Wilcoxon-Kruskal-Wallis test).

Seventeen adult control subjects, 46 adult FRDA patients, and 11 FRDA children performed follow-up evaluations at 1 year interval (11.6 ± 1.6 months). Children had a mean age at onset of 9.5 ± 2.9 years and disease duration of 5.9 ± 1.9 years. In adult patients age at onset was 14.0 ± 4.9, and disease duration was 13.5 ± 7.1 years.

Concomitant medical conditions observed in adult patients were arterial hypertension (n.4); hypercholesterolemia (n.4); hypo- or hyperthyroidism (n.2); psoriatic arthritis (n.1) and Crohn disease (n.1). A 14 year-old FRDA patient had diabetes mellitus. All patients, except one, were homozygous for the presence of GAA expansion on both *FXN* alleles: mean length of shorter allele (GAA1) was 787 ± 186 triplets in children and 653 ± 203 triplets in adult patients. One adult patient was compound heterozygous for GAA expansion (830 triplets) and c.157delC variant on the other *FXN* allele (p.R53AfsX75). *FXN* mRNA was significantly reduced in FRDA patients, being ∼25% of controls (*P* = 0.0001) (Table 1).

**Figure 1:**
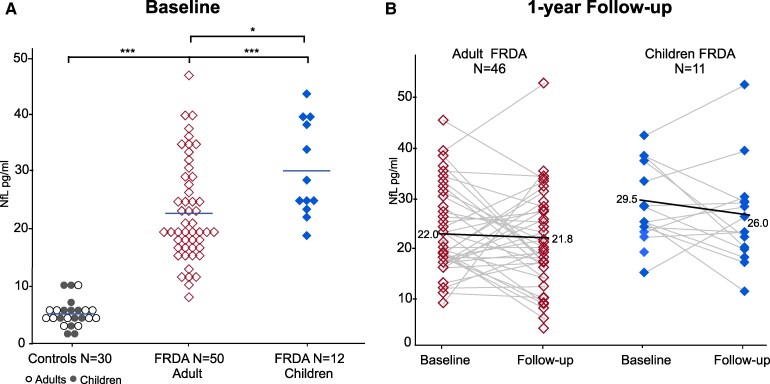
**NfL concentrations in Friedreich ataxia patients.** Graph (**A**) shows baseline NfL levels in FRDA patients and controls individuals. Patients had higher NfL than age–matched controls (***P < 0.001; Wilcoxon test). Children with FRDA had increased plasma NfL levels compared with adult FRDA patients (*P < 0.05; Kruskal-Wallis test). Graph (**B**) shows individual trajectories for longitudinal assessment of NfL in both children and adult FRDA. No significant difference between baseline and 1-year follow-up were observed (paired Wilcoxon test for repeated measures).

NfL plasma concentrations were higher in children with FRDA (28.5 ± 7.7 pg/mL) in comparison with both age-matched control (5.3 ± 2.7 pg/mL; *P* < 0.001) and adult FRDA patients (21.6 ± 8.1; *P* < 0.04) (Table 1; Fig. 1A). Adult patients had also higher NfL levels in comparison with controls. No difference was found in NfL level between children and adult controls (Fig. 1A).

At 1-year follow-up, NfL levels showed no significant changes in the groups of adult FRDA patients (−0.15 pg/mL; −0.7%) and in controls. In children with FRDA we observed a trend toward NfL reduction, with a mean change at 1-year follow-up of −3.5 pg/mL (−11.9%; *P* = 0.265) (Figure 1B).

SARA and ADL scores significantly increased in both adult patients and in children. In adults, SARA increased 1.30 point (*P* < 0.0001) and ADL of 0.83 point (*P* = 0.006). In children, SARA increased 1.25 point (*P* = 0.05) and ADL 2.7 points (*P* = 0.006). mFARS score did not significantly change between baseline and follow-up.

### Correlations between clinical, biochemical and genetic measures

**Figure 2:**
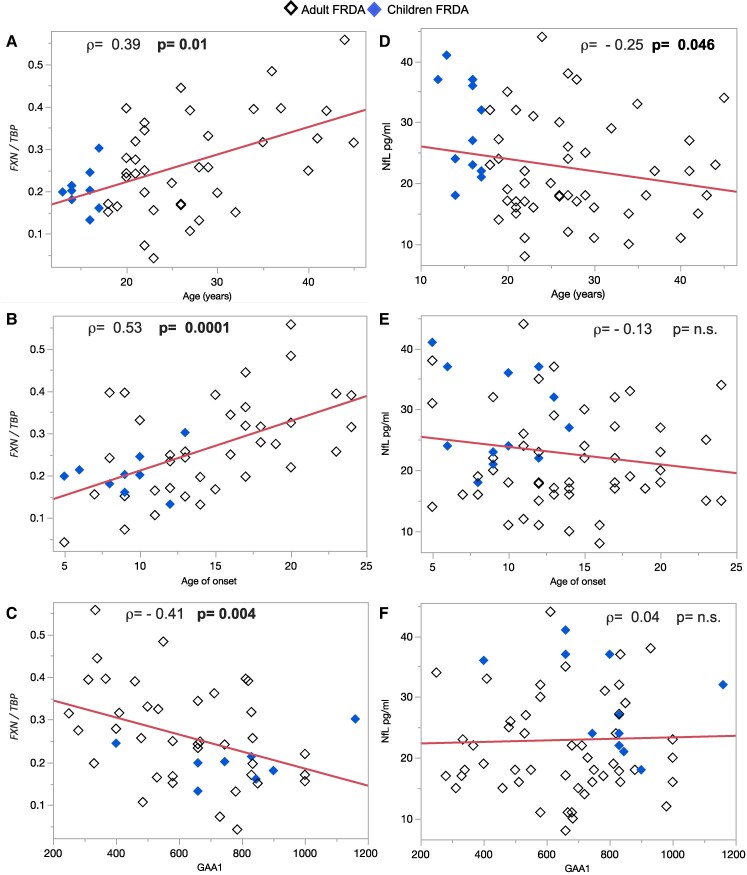
**Frataxin expression and NfL levels as function of age, AOO, and GAA1 repeat.** (A–C) Shows the correlations between frataxin (*FXN*) expression and (**A**) age at examination; (**B**) AOO, and (**C**) GAA1 repeat numbers. All correlations with *FXN* expression were statistically significant (non-parametric Spearman *ρ* correlation). *FXN/TBP*: mRNA of frataxin protein normalized by mRNA of TBP. (**D–F**) show the correlations between NfL plasma levels and the same clinical and genetic measures. A significant inverse correlation was found between plasma NfL levels and age at baseline (*ρ* = −0.255; *P* = 0.046; panel D), while age at onset and GAA1 did not correlate with *FXN* (non-parametric Spearman *ρ* correlation; panels E, F).

We confirmed in our cohort the well-established inverse correlations between GAA1 repeat size and age of onset (AOO) (*ρ* = −0.38; *P* = 0.002). Disease duration significantly correlated with clinical measures of disease severity: ADL (*ρ* = 0.71), SARA score (*ρ* = 0.67), and mFARS score (*ρ* = 0.63) (*P* < 0.0001 for all). FXN expression directly correlated with age (*ρ* = 0.39; *P* = 0.01) and AOO (*ρ* = 0.53; *P* = 0.0001), and inversely correlated with GAA1 (*ρ* = −0.41; *P* = 0.004) (Fig. 2A–C).

In addition, FXN expression was inversely correlated with the clinical scores of ADL (*ρ* = −0.33; *P* = 0.02), SARA (*ρ* = −0.36; *P* = 0.01), and mFARS (*ρ* = −0.37; *P* = 0.01). No correlation was found between FXN and disease duration.

NfL plasma concentrations were inversely correlated with the age of the patients (*ρ* = −0.255; *P* = 0.046, while no statistical correlations were observed between NfL and AOO or GAA1 repeats (Fig. 2D–F).

NfL was not correlated with FXN expression. Furthermore, no correlations were found between NfL levels and any of the clinical variables, including disease duration, ADL, SARA, and mFARS scores.

## Discussion

NfL is an indicator of axonal damage,^18,19^ and it has been extensively studied as a potential biomarker for disease progression, therapeutic response, and safety.^5,20,21^

In Huntington disease, for example, elevated NfL levels might allow accurate distinction between premanifest and juvenile-onset Huntington disease and between people with premanifest Huntington disease and healthy individuals. Moreover, NfL increases with disease progression and correlates with striatal atrophy.^22,23^ In genetic ataxias as Spinocerebellar ataxia type 3 (SCA3), NfL concentrations increase with disease severity and positively correlate with SARA score, while in other type of SCAs, such as SCA1, SCA2 and SCA7, correlation between NfL and disease status was not observed.^6^

Increased NfL has consistently been demonstrated in FRDA patients,^10-14^ and we confirmed this finding in our population. In terms of raw concentration, our values are in line with those previously reported, though obtained with a different analytical technology. In fact, we assessed plasma NfL concentrations by the use of the Simple PlexTM Ella (Ella^TM^) microfluidic platform, while the majority of previous studies assessed either plasma or serum NfL using the Single Molecular Array (Simoa^TM^, Quanterix).^10-13^ In a single study the ELISA technique was used^14^ (Table 2).

**Table 2:** Literature review for NfL blood measurements in patients with FRDA

First author (year)			Comments
Study participants	Healthy controls	FRDA patients	
Zeitlberger^12^ ^,b^	**Single time-point study design**		
N.	13	33	Only adult patients enrolled. NfL higher in FRDA than in controls (*P* < 0.001). No correlations between NfL and age, and measures of disease severity
Age	37 (29–42.5)	34 (23.5–40)	
AOO	NA	13 (7.5–22.5)	
*Plasma NfL*	7.61 (6.2–11.2)	17.10 (13.0–24.4)	
*Simoa^TM^ Quanterix*			
Johnsson^13^	**Single time-point study design**		
N.	-	5	Five adult patients enrolled. Three out of five patients had increased NfL compared with controls. The two oldest and most severely affected patients had normal NfL values
Age (years)		29; 33; 40; 55; 57	
AOO		14; 13;14; 15; 15	
*Plasma NfL* ^a^	< 9	12;14;16;11;13	
*Simoa^TM^ Quanterix*			
Stovickova^14^	**Single time-point analysis**		
N.	17	34	Children and adult patients enrolled.
Age (range)	30 ± 13(13–59)	31 ± 12 (7–55)	NfL levels higher in FRDA than controls (*P* < 0.0001).With increasing age, there is a statistically significant decrease in NFL levels
No. children	4	7	
AOO	-	12 ± 6 (1–33)	
*Serum NfL*	6.1 ± 2.4 (1.9–9.8)	17.7 ± 10.4 (0.7–50.9)	
*ELISA RUO-kit*			
Clay^10,b^	**Longitudinal study design**		Children and adult patients enrolled. NfL higher in FRDA than controls (*P* < 0.0001). NfL inversely correlated with age (Rs = − 0.63, *P* < 0.001). Significant negative correlations with AOO, disease duration, rating scales
N. Cohort ^A^-Cohort ^B^	19^A^–40^B^	85^A^–4^B^	
Age^A+B^ (range)	30.9 ± 16.1 (8–61)	30.7 ± 16.1 (8–85)	
N. children^A+B^	15	46	
AOO	NA	13.0 ± 6.4	
*Serum NfL*	[7.9 ± 4.5]^A^[4.7 ± 3.0]^B^	[18.2 ± 9.4]^A^[24.8 ± 14.6]^B^	**NfL stable at 14-month follow-up (*N*** **=** **32) (+3%)**
*Simoa^TM^ Quanterix*			
Hayer^11,b^	**Longitudinal study design**		
N.	30	99	Children and adult patients enrolled.
Age (range)	45.3 ± 14.1 (18–68)	38.4± 13.05 (16–68)	High NfL levels in patients aged 16–47, but not in patients aged ≥ 48 years. NfL inversely correlated with GAA1 repeat length, but not with age, ataxia rating scale, severity, disease duration, and AOO.
No. children	1	8	
AOO	NA	NA	
*SerumNfL*	21.2 (4–49)	26.1 (0–78)	**NfL stable at 24-month follow-up (*N*** **=** **14) (**34.1; range 11–81)
*Simoa^TM^ Quanterix*			
Present study (2025)	**Longitudinal study design**		
N.	30	62	Both children and adult patients enrolled. Higher NfL in children compared with adult patients (*P* < 0.02). Negative correlation between age and NfL concentrations (*P* = 0.046).
Age (range)	21.9 ± 6.5 (12–38)	25.1 ± 8.5 (12–45)	
N. children	12	12	
AOO	-	13.1 ± 4.8	
*Plasma NfL*	5.9 ± 3.6	22.9 ± 8.5	**NfL stable at 12 month follow-up (*N*** **=** **56) (**22.6; range 19–26)
*Ella assay kit*			

Data are reported as mean ± SD, or as median and range/interquartile range into parenthesis.

^a^NfL concentrations are reported as pg/mL. AOO, Age of onset (years); ELISA, Enzyme-linked Immunosorbent Assay; NA, not available.

^b^These studies are included in meta-analysis performed by Peng *et al*.^6^ (Mov Disord, 2022) See References.

Ella assay kit (ProteinSimple, CA, USA) on Ella device (ProteinSimple, CA, USA).

The comparison between Simoa^TM^ and Ella^TM^ immunoassays demonstrated that the results obtained with the two technologies are strongly correlated (*ρ* = 0.86, *P* < 0.0001), and that both platforms offer excellent sensitivity for the use in clinical practice.^24^ However, it should be mentioned that the absolute values of NFL measured by Simoa^TM^ are globally lower that the values measured by Ella^TM^ and therefore the assay methodology should be considered when interpreting absolute NfL values in cross-study comparisons.^24^

We showed that NfL plasma concentrations are higher in children with FRDA both compared with age-matched healthy controls and compared with adult FRDA patients (≥18 years) (Fig. 1A).

In physiological conditions, children have elevated NfL level after birth, then the concentration decreases by ∼6.8% per year until the age of 10.^25^ In young and adult individuals NfL remains stable for several decades, and it increases in elderly people ≥60 years.^6^ The high NfL concentration in the first decade of life has been associated to the growth and maturation of central nervous system.^26^

In our study, we avoid the bias of physiological NfL changes, by the selection a cohort of patients aged between 12 and 45 years, when NfL levels are stable. The trend of NfL observed in FRDA differs from that described in adult-onset disorders, as concentrations do not increase with disease progression, but are high in proximity of disease onset and decrease in more advanced disease stages.

A similar pattern of neurofilament dynamics has been observed in other pediatric neurodegenerative diseases. In spinal muscular atrophy (SMA), for example, both NfL and phosphorylated neurofilament heavy subunit (pNfH) are elevated at disease onset and show an age-related decrease.^27^

Significant inverse correlations between NfL and AOO, disease duration and the clinical measures of disease severity were observed in both SMA^27^ and in FRDA.^10^

We found that age but not disease severity was the major determinant for NfL blood concentrations.

Our results are in line with other studies in FRDA, in which NfL and age were consistently proven to be related, while no correlations with disease progression or clinical scores were observed.^10,14^ In particular, correlation with age was found in cohorts including both pediatric and adult patient populations, while in cohorts mainly composed of adult FRDA patients, NfL values are higher than in controls, but do not correlate with age.^11,12^ Interestingly, Johnsson *et al*.^13^ describe NfL concentration similar to healthy controls in two severely affected FRDA patients, aged 55 and 57, with a disease duration of 40 and 42 years.^13^

To explain this finding, it has been hypothesized that NfL is high when important damage of central and peripheral nerve axons occurs and both degenerative and regenerative processes might coexist.^28^ In later FRDA disease phases, NFL is maintained stable matching a constant neuronal degeneration, and then it declines in the very late stage when neuronal loss prevails.^28^

Our study defines the distinctive NfL profile over time of FRDA disease and confirms that neurofilament represents one of the most informative and early indicators of axonal damage.

NfL may also play a potential role as pharmacodynamic biomarker in response to therapeutic interventions, as demonstrated in SMA following treatment with nusinersen (Spinraza).^29^ This may be particularly relevant in pediatric populations where functional measures are less sensitive or disease evolution occurs more slowly.^30^

## Data Availability

The data that support the findings of this study are available from Open Repository of the Fondazione IRCCS Istituto Neurologico Carlo Besta (https://zenodo.org/communities/besta) upon reasonable request to the corresponding author. The data are not publicly available as they contain information that could compromise the privacy of research participants.
